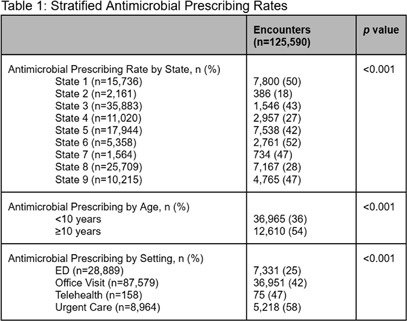# Setting, Age, and Geography Impact Antimicrobial Prescribing in Children with Upper Respiratory Infections Across a Large Health System

**DOI:** 10.1017/ash.2025.271

**Published:** 2025-09-24

**Authors:** Reese A. Cosimi, Melinda Mackey, Maria Mupanomunda, Julie Hunt, Ashlin Jones, Aaron Shoemaker, Stacy Garrett-Ray, Frederick A. Masoudi, Thomas Aloia, Allison Bollinger, Mohamad Fakih

**Affiliations:** 1 Ascension, St. Louis, MO; 2 Ascension Data Sciences Institute, Ascension, St. Louis, MO; 3 University of Maryland School of Medicine, Baltimore, MD; 4 Wayne State University School of Medicine, Detroit, MI

## Abstract

**Background:** Upper respiratory infections (URIs) are common causes of outpatient visits in children. While many URIs are viral, antimicrobial prescribing remains high. In preparation for action planning to address this issue within our multi-state health system, this study aimed to characterize current antimicrobial prescribing patterns for pediatric URIs in our outpatient setting. **Methods:** Retrospective analysis of pediatric (<18 years) antimicrobial prescribing for URI diagnosis codes in 639 outpatient sites (nine states), including clinics, urgent cares, and emergency departments (ED) between July 1, 2023 to June 30, 2024. Primary outcome was overall antimicrobial prescribing rates for URIs and by individual URI diagnosis (sinusitis, bronchitis, pharyngitis, otitis media). Logistic regression machine learning model was used with SHapley Additive exPlanations (SHAP) analysis to show feature contributions to antimicrobial prescribing. **Results:** A total of 125,590 patient visits by children with URI were included. Antimicrobial prescribing rates varied by diagnosis (sinusitis: 53%, bronchitis: 18%, pharyngitis: 45%, otitis media: 40%, p<0.001). Overall prescribing ranged from 18%-52% across states. Patients seen in the ED had the lowest use of antimicrobials (25%) while those seen in urgent care had the highest utilization (58%). Non-bronchitis diagnosis, non-ED encounters, ≤10 years of age, and specific states had the strongest positive associations with antimicrobial prescribing, while race and social vulnerability index (SVI) were not associated. **Conclusions:** Antimicrobials were most commonly prescribed for pediatric patients seen for sinusitis, pharyngitis, and otitis media. Factors most associated with increased prescribing included non-ED encounters, >10 years of age, and geography. These data support action to standardize practices and address clinical variation.

**Figure:**
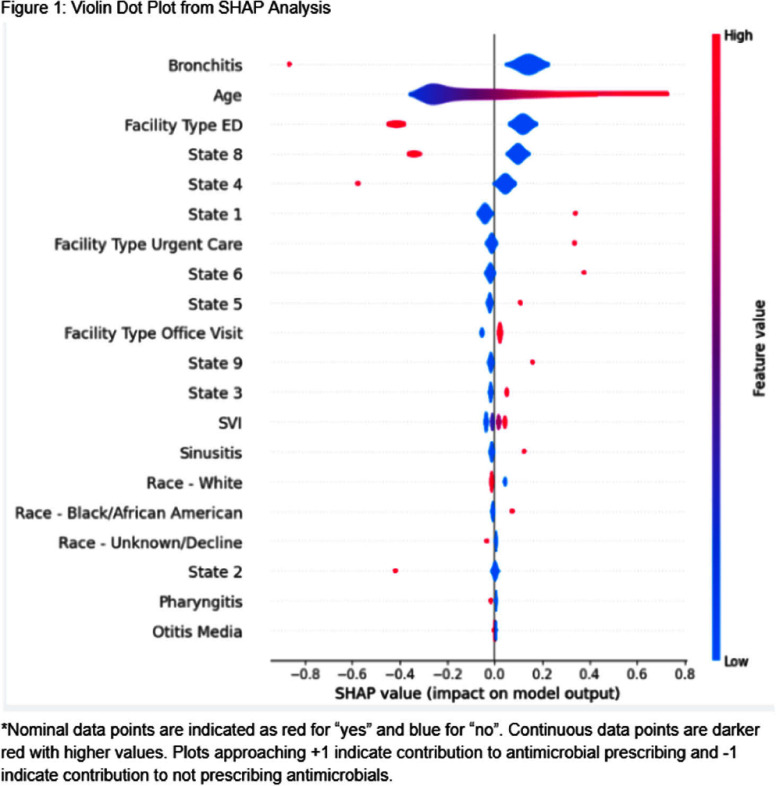

**Figure:**